# Evaluation of a lateral flow immunoassay for the quantitative measurement of immunoglobulin G in foals compared with radial immunodiffusion

**DOI:** 10.1093/jvimsj/aalag216

**Published:** 2026-09-21

**Authors:** Yongwoo Sohn, Sung Jun An, Han Sang Oh, Jungho Kim, Eliot Forbes, Seung-Ho Ryu

**Affiliations:** Veterinary Department, Korea Racing Authority, Gwacheon 13822, Republic of Korea; Department of Artificial Intelligence, Cheju Halla University, Jeju 63092, Republic of Korea; R&D Center, BioNote, Inc, Hwaseong-si, Gyeonggi-do 18449, Republic of Korea; R&D Center, BioNote, Inc, Hwaseong-si, Gyeonggi-do 18449, Republic of Korea; Racing Integrity Board, Greenlane, Private Bag 17902, Auckland 1546, New Zealand; Department of Equine Resources Science, Cheju Halla University, Jeju 63092, Republic of Korea

**Keywords:** foal, immunoglobulin G, lateral flow immunoassay, passive immunity

## Abstract

**Background:**

Reliable point-of-care diagnostic tests are essential for diagnosing failure of transfer of passive immunity (FTPI) in foals. Quantitative lateral flow immunoassays (LFIAs) could reduce subjectivity but have not been well validated in foals.

**Hypothesis/Objectives:**

To validate the performance and diagnostic accuracy of a LFIA for quantifying immunoglobulin G (IgG) concentrations in foals, using radial immunodiffusion (RID) as the reference standard.

**Animals:**

Forty-two neonatal foals were sampled within 24 h of birth.

**Methods:**

This prospective exploratory diagnostic accuracy study evaluated the diagnostic accuracy of an LFIA for neonatal foal IgG measurement in accordance with the STARD guidelines. Blood samples were analyzed by LFIA, RID, and a SNAP-based ELISA. Primary analyses used serum samples. Diagnostic performance was evaluated for serum, plasma, and whole blood at predefined RID thresholds (<400 and ≤ 800 mg/dL) using receiver operating characteristic analysis, sensitivity, specificity, and Bland–Altman analysis.

**Results:**

Lateral flow immunoassays results were strongly correlated with RID (*R*^2^ = 0.91) within the analytical range (100-1000 mg/dL). The assay demonstrated good linearity, precision, and accuracy. Receiver operating characteristic analysis showed high point estimates for diagnostic performance across sample matrices, with AUCs ranging from 0.988 to 1.000, although estimates were imprecise because of the limited number of FTPI-positive foals.

**Conclusions and clinical importance:**

Lateral flow immunoassays demonstrated good agreement with RID for FTPI classification and could be suitable as a point-of-care screening tool, but is not interchangeable with RID for quantitative measurement. Although the limited number of FTPI-positive foals warrants cautious interpretation, these findings support LFIA as a screening tool for FTPI classification.

## Introduction

Early and accurate detection of failure of transfer of passive immunity (FTPI) is important in neonatal foals, because inadequate passive immunity increases susceptibility to severe infection.^1–4^ Failure of transfer of passive immunity is a recognized risk factor for sepsis, omphalophlebitis, septic arthritis, and respiratory disease and is associated with increased morbidity and mortality in foals.^4–8^ Neonatal foals experience declining immunoglobulin absorption within the first 12-24 h after birth, so early ingestion of colostrum is vital.^2^ Based on serum immunoglobulin G (IgG) concentrations, foals are categorized as: < 400 mg/dL (complete FTPI), 400-800 mg/dL (partial FTPI), and > 800 mg/dL (adequate transfer of passive immunity).^9^ The relationship between FTPI and neonatal sepsis remains debated because FTPI is often considered a predisposing rather than essential factor and some foals develop diarrhea and bacteremia despite adequate IgG concentrations.^10^ For critically ill foals, early prognostic assessment can also guide treatment decisions, since survival prediction models such as the Foal Survival Score (FSS) incorporate clinical and laboratory variables, including IgG concentration.^11–14^ Accurate and timely IgG measurement is therefore central to both FTPI screening and prognostic assessment.

Several methods are available for assessing passive immunity in neonatal foals, including direct measurement of serum IgG concentration, serum total protein (TP), refractometry and semiquantitative stall-side assays. Although TP measurement and refractometry offer practical advantages in field settings, they are indirect surrogate markers and can be influenced by factors unrelated to immunoglobulin status, such as hydration status or acute phase responses.^15^^,^^16^ In contrast, direct measurement of serum IgG provides a more specific assessment of passive immunity and remains the basis for defining FTPI categories. Previous studies comparing IgG-based assays with alternative screening methods have highlighted trade-offs between analytical accuracy, turnaround time, and clinical practicality, underscoring the need for rapid, quantitative point-of-care IgG assays with objective readouts.^15^^,^^16^ Semiquantitative assays that report IgG concentrations in broad categorical ranges could make it harder to distinguish foals with borderline FTPI and could limit their usefulness for monitoring changes in IgG concentration over time.

Radial immunodiffusion (RID) is widely regarded as the reference method for IgG quantification but it requires 18-24 h to complete and is labor intensive.^7^^,^^17^ The SNAP foal IgG test (IDEXX Laboratories) is a common ELISA used in practice but is semiquantitative and its interpretation can be subjective.^18^ Quantitative lateral flow immunoassays (LFIAs) provide rapid numerical results and could improve decision making at the point of care by delivering timely IgG concentrations and reducing interpretive subjectivity.^19^

This study aimed to validate the analytical performance and diagnostic accuracy of a quantitative LFIA for field-based IgG measurement in neonatal foals. Radial immunodiffusion was used as the reference standard for all primary analytical and diagnostic performance evaluations, whereas the SNAP-based ELISA was included solely as a secondary comparator to reflect commonly used field-based testing methods and provide additional clinical context.

## Materials and methods

This exploratory diagnostic accuracy study was designed and reported in accordance with the STARD guidelines.^20^ Forty-two Thoroughbred foals with no apparent clinical abnormalities at the time of blood collection from 18 commercial breeding farms were enrolled within 24 h of birth. Blood samples were collected during routine veterinary visits with owner consent. Serum was used for primary method-comparison analyses because RID, the reference standard, requires serum samples. Whole blood and plasma were additionally evaluated to assess point-of-care applicability across clinically relevant sample matrices.

Blood was collected into serum-separating and EDTA tubes. Serum and plasma were separated by centrifugation and stored at −80°C until analysis. Immunoglobulin G concentrations were measured using a fluorescence-based LFIA (Vcheck foal IgG test, BioNote Inc, Korea), RID (Triple J Farms, USA), and a commercially available SNAP-based ELISA (IDEXX Laboratories, USA). The SNAP-based ELISA was included as a secondary comparator to provide clinical context but was not used as the reference method. The LFIA and RID testing were performed according to the manufacturers’ instructions, and RID measurements were interpreted by laboratory personnel blinded to LFIA and ELISA results.

Testing with the LFIA was performed using serum, plasma, or whole blood. The analytical range of the assay was 100-1000 mg/dL; samples outside this range were excluded from primary agreement analyses. Clinically relevant IgG thresholds were predefined as < 400 mg/dL (complete FTPI) and ≤ 800 mg/dL (partial or complete FTPI). Radial immunodiffusion was used as the reference standard for all primary analytical and diagnostic performance evaluations.

Analytical validation included assessments of linearity, intra-assay precision, inter-assay precision, and method agreement. Accuracy and agreement between LFIA and RID were evaluated using Pearson correlation, linear regression, Bland–Altman analysis, and Cohen’s κ statistics. Diagnostic performance was assessed using receiver operating characteristic (ROC) curve analysis with calculation of sensitivity, specificity, and area under the curve (AUC). Analyses were performed separately for serum, plasma, and whole blood datasets, with one observation per foal included within each matrix-specific analysis to minimize bias related to repeated measurements.

Statistical analyses were performed using Python (version 3.7.12), with significance set at *P* < .05. Confidence intervals for sensitivity and specificity were estimated using the Wilson method and ROC AUC CIs were estimated using the DeLong method. Detailed descriptions of animal enrollment, sample handling, assay procedures, statistical methods, and extended agreement analyses are provided in the Appendix.

## Results

**Figure 1:**
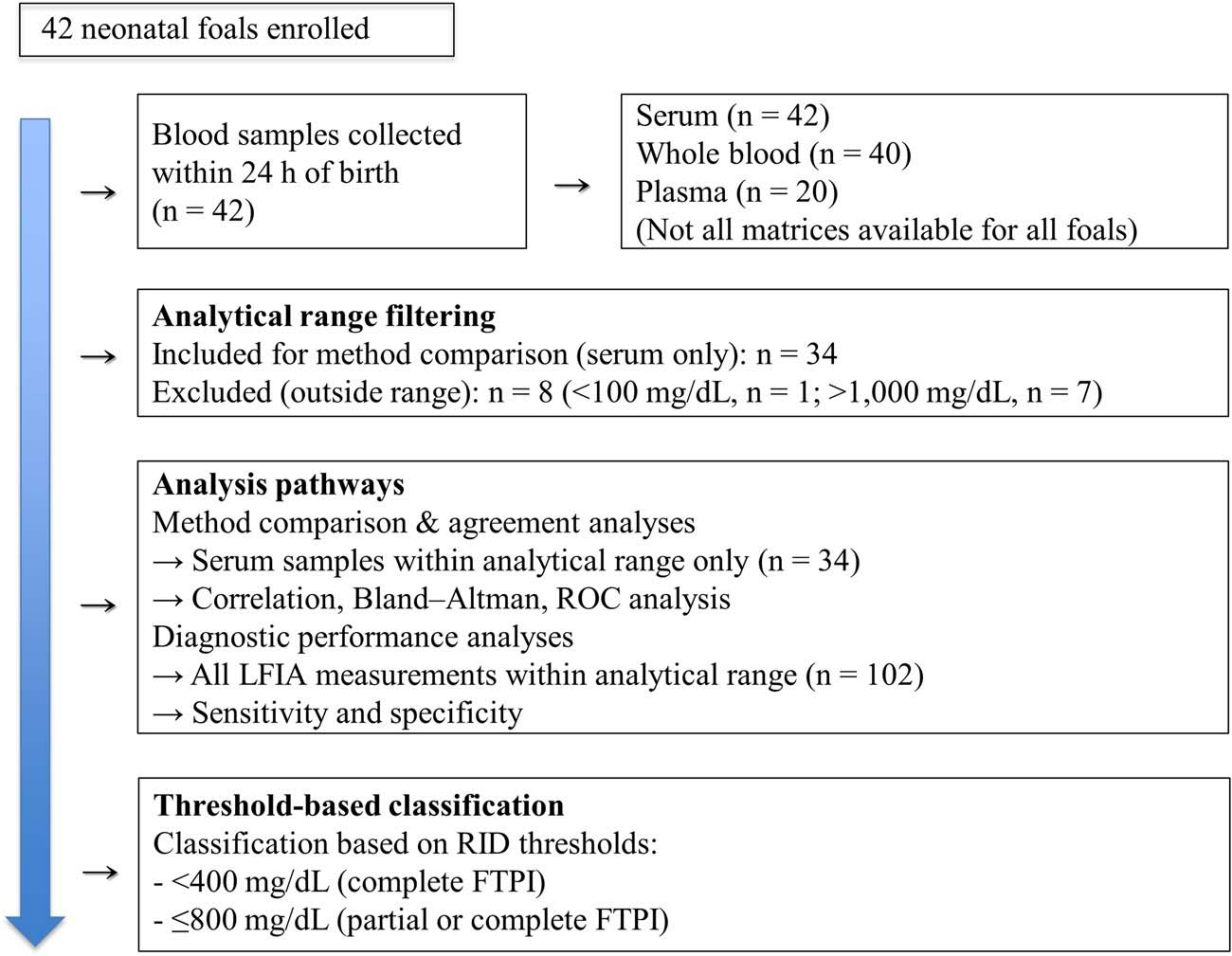
Study design and sample flow. A total of 42 neonatal foals were enrolled, and blood samples were collected within 24 h of birth. Serum, plasma, and whole blood samples were analyzed using an LFIA, yielding 102 measurements (serum, *n* = 42; whole blood, *n* = 40; plasma, *n* = 20). RID, the reference standard, was performed on serum samples only. Accordingly, method comparison and agreement analyses were restricted to serum samples within the analytical range (100-1000 mg/dL) (*n* = 34). ROC analyses were performed separately for each sample matrix (serum, whole blood, and plasma) to reflect matrix-specific diagnostic performance. Diagnostic performance analyses were conducted separately for each sample matrix, with one measurement per foal per matrix. No pooling across sample types was performed. Abbreviations: LFIA = lateral flow immunoassay; RID = radial immunodiffusion; ROC = receiver operating characteristic.

A total of 42 serum samples, 40 whole blood samples and 20 plasma samples were obtained from the enrolled foals and included in the matrix-specific analyses (Figure 1). Because the ELISA was included solely as a secondary clinical comparator, no formal diagnostic performance or analytical comparison between the LFIA and ELISA was performed.

### Linearity assessment

**Figure 2:**
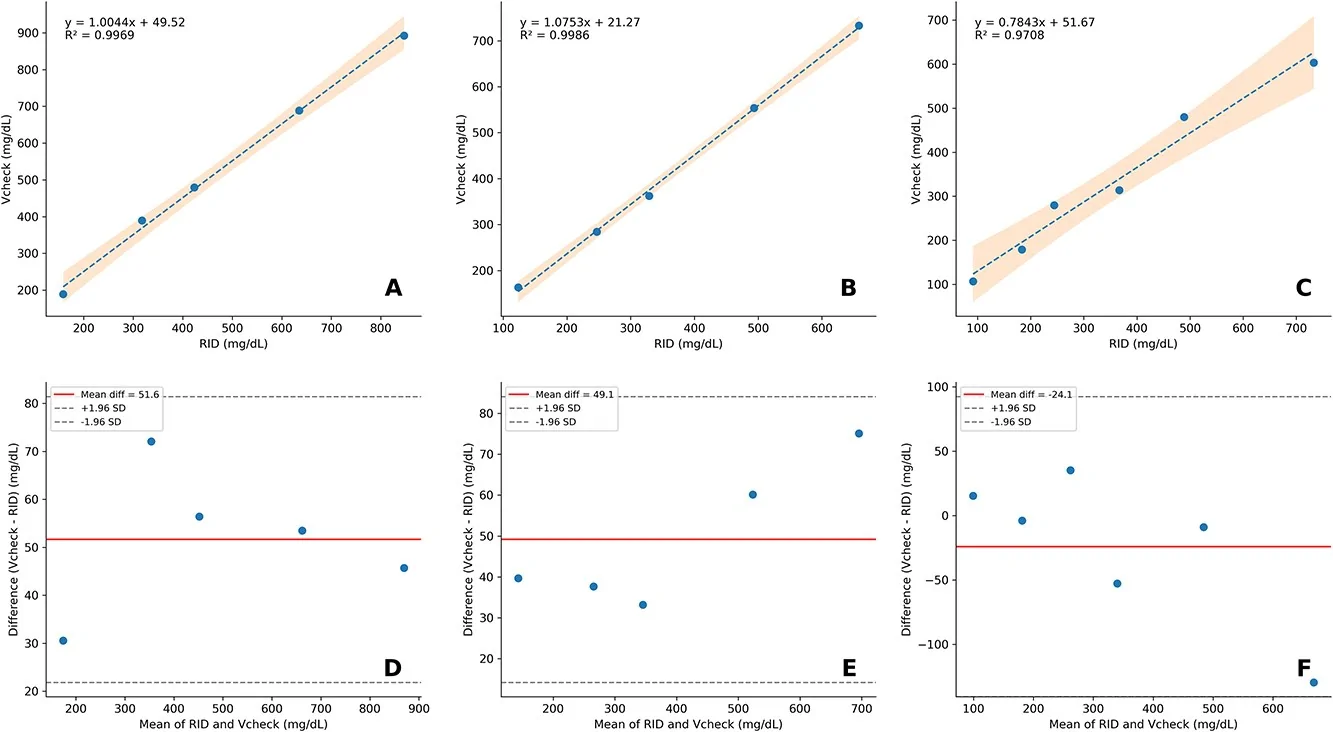
Linearity assessment of the LFIA. Serial dilutions of 3 serum samples with high IgG concentrations were evaluated across the analytical range of 100-1000 mg/dL. Linear regression plots (A–C) compare expected IgG concentrations and LFIA-measured values and corresponding Bland–Altman plots (D–F) assess agreement across dilution levels. Panels A and D, B and E, and C and F represent matched analyses of the same samples. Abbreviations: IgG = immunoglobulin G; LFIA = lateral flow immunoassay.

A strong linear relationship was observed between expected IgG concentrations and LFIA-measured values across the analytical range of 100-1000 mg/dL. Linear regression analyses of the individual samples yielded coefficients of determination (*R*^2^) of 0.9708, 0.9969, and 0.9986, indicating strong assay linearity. No significant deviation from linearity was observed across the dilution series for any of the 3 samples (Figure 2).

### Precision analysis

For the LFIA, the intra-assay coefficient of variation (CV) ranged from 0.75% to 4.30%, with a mean CV of 1.97%. The inter-assay CV ranged from 1.84% to 4.08%, with a mean CV of 3.02%. All CV values were well below the accepted threshold of 25% for bioanalytical assays, indicating good repeatability and reproducibility across clinically relevant IgG concentrations. Intra-assay and inter-assay CVs for each concentration level are summarized in Table 1.

**Table 1:** Precision analysis of the LFIA at 4 IgG concentration levels.

IgG concentration (mg/dL)	Intra-assay CV (%)	Inter-assay CV (%)
**226**	4.30	2.90
**432**	1.58	3.27
**775**	1.25	4.08
**1005**	0.75	1.84
**Mean**	1.97	3.02

Abbreviation: CV = coefficient of variation; IgG = immunoglobulin G; LFIA = lateral flow immunoassay

Intra-assay CV was calculated from 3 replicate measurements per day across 5 nonconsecutive days (*n* = 15 per sample). Inter-assay CV was calculated using daily mean values across 5 days (*n* = 5 per sample).

### Reference standard reliability

Inter-reader reliability of RID measurements was excellent, with an ICC of 0.998 (95% CI, 0.992-1.000). Intra-assay repeatability also demonstrated excellent agreement, with an ICC of 0.997 (95% CI, 0.987-1.000). These results indicate high consistency and minimal reader-dependent variability in the RID reference standard.

### Accuracy and agreement (method comparison)

A total of 42 neonatal foals were enrolled in the study. Of these, 34 serum samples yielded LFIA results within the analytical range of 100-1000 mg/dL and were included in primary method comparison and agreement analyses. The remaining samples fell outside the analytical range of the LFIA, including 1 sample with a value < 100 mg/dL and 7 samples with values > 1000 mg/dL.

**Figure 3:**
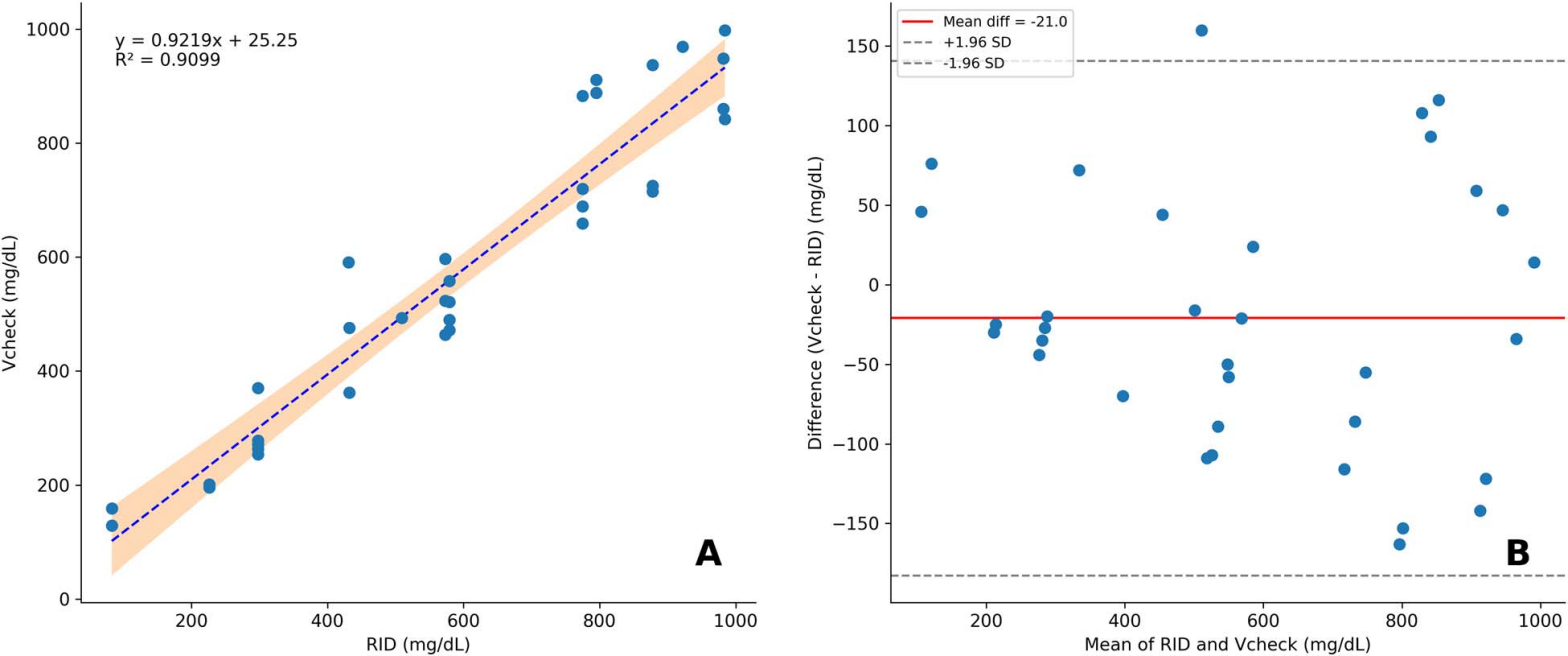
Accuracy and agreement between the LFIA and RID for measurement of serum IgG concentrations in neonatal foals. (A) Linear regression analysis with 95% CIs compares LFIA-measured and RID-measured IgG concentrations. (B) Bland–Altman plot illustrates the difference between LFIA and RID measurements plotted against their mean, with the solid line indicating the mean bias and dashed lines representing the 95% limits of agreement. Abbreviations: IgG = immunoglobulin G; LFIA = lateral flow immunoassay; RID = radial immunodiffusion.

Accuracy of the LFIA was evaluated by comparing LFIA and RID measurements within the analytical range of 100-1000 mg/dL. Linear regression demonstrated a strong association between LFIA and RID measurements, yielding LFIA = 0.9219 × RID + 25.25 (*R*^2^ = 0.9099) (Figure 3A). The slope was not significantly different from 1.0 (*P* = .1378), indicating no significant proportional bias.

Bland–Altman analysis showed a mean bias of −21.0 mg/dL, indicating a slight underestimation of IgG concentrations by LFIA relative to RID (Figure 3B). The 95% limits of agreement ranged from −182.6 to 140.7 mg/dL, with the majority of differences falling within these limits and no evidence of systematic bias across the measurement range. Larger negative differences were observed at higher IgG concentrations (>800 mg/dL). These findings support use of the LFIA for FTPI classification but do not support numerical interchangeability with RID.

**Figure 4:**
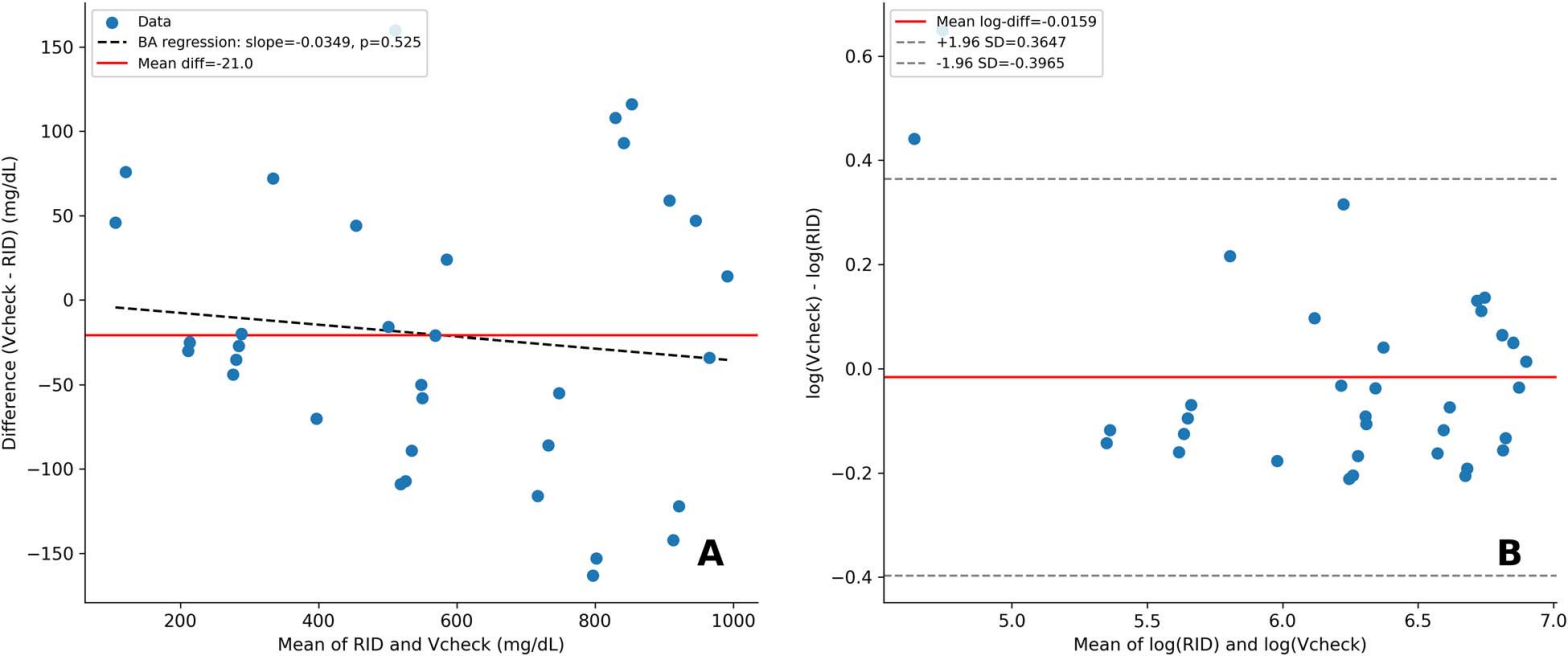
Extended Bland–Altman analyses assessing proportional agreement between LFIA and RID. (A) Bland–Altman plot with regression of LFIA–RID differences on the measurement mean assesses proportional bias. (B) Log-transformed (ratio-based) Bland–Altman plot demonstrates agreement across the IgG concentration range. Abbreviations: IgG = immunoglobulin G; LFIA = lateral flow immunoassay; RID = radial immunodiffusion.

Regression of LFIA–RID differences on the measurement mean did not demonstrate evidence of proportional bias on the raw scale (slope = −0.035, *P* = .53; Figure 4A). Log-scale Bland–Altman analysis suggested modest proportional bias at higher IgG concentrations (*P* = .02; Figure 4B); however, the average ratio bias remained small (LFIA/RID = 0.98), indicating minimal systematic proportional error.

In addition to continuous measures of agreement, categorical agreement between LFIA and RID was assessed using 3 clinically relevant IgG categories (<400, 400-800, and > 800 mg/dL). Categorical agreement between LFIA and RID was substantial, with an unweighted Cohen’s κ of 0.855 (95% CI, 0.742-0.967). Because IgG concentration categories are ordinal, weighted κ values were also calculated, yielding κ = 0.892 with linear weighting and κ = 0.929 with quadratic weighting. Based on RID measurements, 2 foals were classified as < 400 mg/dL, 6 as 400-800 mg/dL, and 26 as > 800 mg/dL among the 34 serum samples included in the categorical agreement analysis.

Of the 34 serum samples included in the categorical agreement analysis, 28 samples were classified into the same IgG category by both methods, whereas 6 samples showed discordant classification. One discordant case occurred near the 400 mg/dL threshold, in which the RID value was 432 mg/dL while the corresponding LFIA measurement was 362 mg/dL, resulting in classification below the cutoff by LFIA. The remaining 5 discordant cases occurred near the 800 mg/dL threshold, with LFIA values both slightly above and slightly below the corresponding RID measurements. These discrepancies were confined to category boundaries rather than reflecting systematic disagreement across the analytical range.

Misclassification was explicitly quantified at clinically relevant thresholds (<400 and ≤ 800 mg/dL). The small number of discordant cases highlights the need for cautious interpretation near decision cutoffs, while overall categorical agreement remained high.

### Sensitivity and specificity

Sensitivity and specificity analyses were performed separately for each sample matrix (serum, whole blood, and plasma), using independent observations within each dataset. Using RID as the reference standard, LFIA diagnostic performance was evaluated at 2 cutoffs (<400 and ≤ 800 mg/dL) within each matrix-specific dataset.

In the serum samples, at the < 400 mg/dL cutoff, sensitivity and specificity were 100% (2/2; 95% CI, 34.2-100.0) and 100% (40/40; 95% CI, 91.2-100.0), respectively. At the ≤ 800 mg/dL cutoff, sensitivity and specificity were 75.0% (6/8; 95% CI, 40.9-92.9) and 97.1% (33/34; 95% CI, 85.1-99.5), respectively.

In the whole blood samples, at the < 400 mg/dL cutoff, sensitivity and specificity were 100% (2/2; 95% CI, 34.2-100.0) and 97.4% (37/38; 95% CI, 86.5-99.5), respectively. At the ≤ 800 mg/dL cutoff, sensitivity and specificity were 87.5% (7/8; 95% CI, 52.9-97.8) and 96.9% (31/32; 95% CI, 84.3-99.4), respectively.

In the plasma samples, at the < 400 mg/dL cutoff, sensitivity and specificity were 100% (5/5; 95% CI, 56.6-100.0) and 100% (15/15; 95% CI, 79.6-100.0), respectively. At the ≤ 800 mg/dL cutoff, sensitivity and specificity were 100% (10/10; 95% CI, 72.2-100.0) and 100% (10/10; 95% CI, 72.2-100.0), respectively.

### Receiver operating characteristic analysis

**Figure 5:**
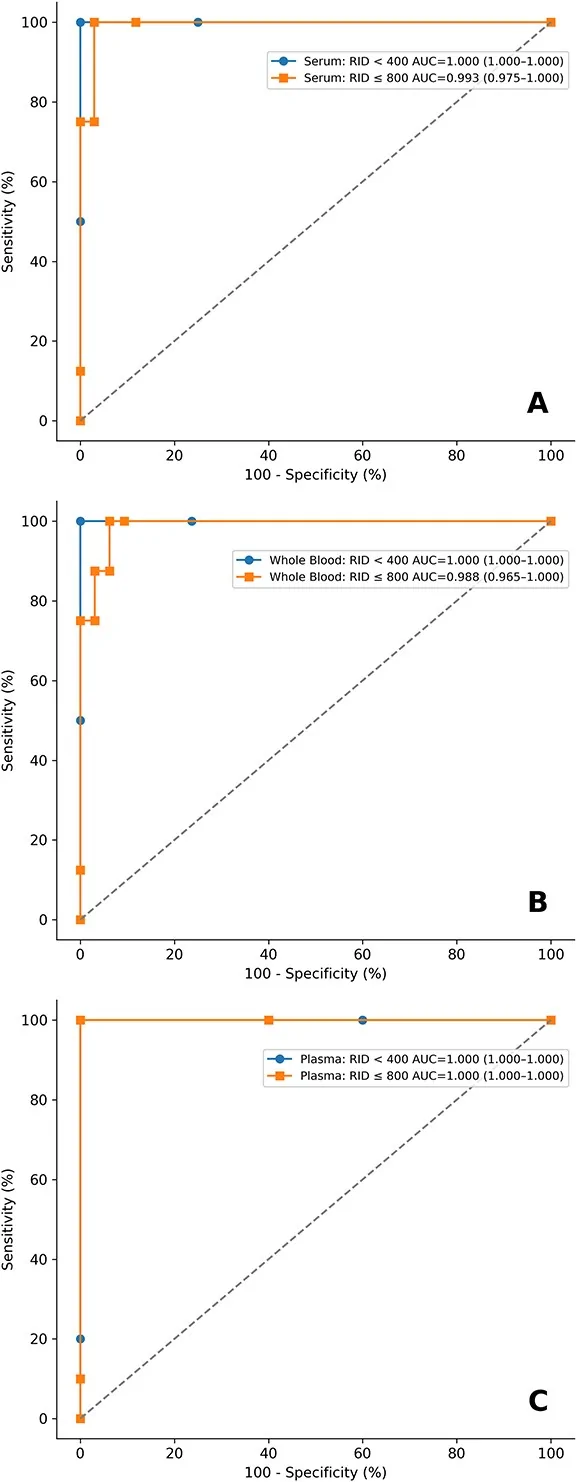
ROC curves of the LFIA for identification of FTPI using RID as the reference standard, stratified by sample matrix. Ninety-five percent CIs were estimated. (A) ROC curves for serum samples. The AUC was 1.000 (95% CI, 1.000-1.000) for RID IgG < 400 mg/dL and 0.993 (95% CI, 0.975-1.000) for RID IgG ≤ 800 mg/dL. (B) ROC curves for whole blood samples. The AUC was 1.000 (95% CI, 1.000-1.000) for RID IgG < 400 mg/dL and 0.988 (95% CI, 0.965-1.000) for RID IgG ≤ 800 mg/dL. (C) ROC curves for plasma samples. The AUC was 1.000 (95% CI, 1.000-1.000) for both RID IgG < 400 mg/dL and RID IgG ≤ 800 mg/dL. Abbreviations: AUC = area under the curve; FTPI = failure of transfer of passive immunity; IgG = immunoglobulin G; LFIA = lateral flow immunoassay; ROC = receiver operating characteristic; RID = radial immunodiffusion.

Receiver operating characteristic analysis was performed to evaluate the diagnostic performance of the LFIA for detection of FTPI using RID as the reference standard (Figure 5). The analysis was performed separately for each sample matrix (serum, whole blood, and plasma) to reflect matrix-specific diagnostic performance.

In serum samples, at the < 400 mg/dL cutoff, the AUC was 1.000 (95% CI, 1.000-1.000), and at the ≤ 800 mg/dL cutoff, the AUC was 0.993 (95% CI, 0.975-1.000).

In whole blood samples, at the < 400 mg/dL cutoff, the AUC was 1.000 (95% CI, 1.000-1.000), and at the ≤ 800 mg/dL cutoff, the AUC was 0.988 (95% CI, 0.965-1.000).

In plasma samples, at both the < 400 and ≤ 800 mg/dL cutoffs, the AUC was 1.000 (95% CI, 1.000-1.000). Area under the curve estimates of 1.000 should be interpreted cautiously because of the small number of positive cases within each matrix-specific dataset.

## Discussion

To minimize potential bias related to repeated measures, primary statistical analyses were restricted to serum samples, thereby limiting the effect of within-animal correlation. Accordingly, the agreement and accuracy results presented here are directly applicable to serum samples, which represent the reference matrix for FTPI assessment.

The LFIA showed strong linearity across the dilution series and good agreement with RID for FTPI classification. Precision and reproducibility were supported by low intra-assay CVs of 0.75%-4.30% comparable to those reported for automated turbidimetric immunoassay (TIA) systems (CV about 3%).^7^ Although inter-assay CVs (1.84%-4.08%) were higher than those reported for automated TIA (1%-4%),^7^ this might reflect the broader analytical range evaluated in this study. Nevertheless, both intra- and inter-assay CVs remained well within the acceptable limit of 25%.^21^ Overall, the LFIA demonstrated good agreement with the reference standard RID across clinically relevant IgG categories.

Several factors can influence assay performance. Small differences in sample handling and test setup could contribute to variability and this effect could increase as procedural steps accumulate.^22^ This likely explains the slightly lower coefficient of variation observed in the intra-assay experiment, in which repeated measurements were performed on the same sample, compared with the inter-assay experiment, in which the entire testing process was repeated multiple times. Importantly, the LFIA performed robustly, suggesting that minor handling differences are unlikely to cause clinically significant errors in IgG measurement.

When assessing FTPI, high sensitivity is critical, as missing FTPI cases can lead to serious clinical consequences.^23^^,^^24^ Conversely, high specificity is important to avoid unnecessary plasma transfusions, which carry a risk of adverse reactions and increased costs.^25^ Consistent with these clinical priorities, ROC analysis showed high point estimates for diagnostic performance at both complete FTPI (<400 mg/dL) and partial or complete FTPI (≤800 mg/dL), although estimates should be interpreted cautiously because of the limited number of FTPI-positive foals.

Given the subjective visual interpretation required for the SNAP-based ELISA, results were independently assessed by 1 field clinician and 2 laboratory researchers to minimize interpretive bias, whereas LFIA measurements were generated using an automated analyzer. The LFIA provides an automated objective numerical result within approximately 5 min, thereby reducing reader-dependent variability and facilitating point-of-care FTPI screening. The SNAP-based ELISA was included to provide clinical context because it is commonly used in field settings; however, it was not intended to serve as the reference method for analytical validation of the LFIA. Accordingly, the primary objective of this study was to validate the LFIA against RID rather than to compare the analytical performance of the LFIA and ELISA. Therefore, ELISA findings were considered descriptive clinical information only and were not used for formal quantitative comparison or analytical validation of LFIA performance. Confidence intervals were estimated using the Wilson method, which is appropriate for small sample sizes; however, the small number of positive cases reduced the precision of these estimates. Because only 2 foals had RID IgG concentrations below 400 mg/dL, sensitivity estimates at this threshold should be interpreted with caution, as the small number of cases resulted in wide CIs. In this context, although the LFIA demonstrated good diagnostic performance overall, the agreement analyses do not support quantitative interchangeability with RID. Although the log-transformed Bland–Altman analysis suggested a tendency for the LFIA to underestimate IgG concentrations at higher values, this proportional bias was not sufficiently consistent across the analytical range to support a simple correction factor or direct numerical conversion between methods. Instead, its primary clinical value lies in its ability to accurately categorize foals according to FTPI status at clinically relevant thresholds rather than to provide exact quantitative equivalents to RID measurements.

This study has limitations. The sample size was modest and sampling was limited to a small number of farms, which might reduce generalizability. Potential confounding factors such as timing and volume of colostrum intake, subclinical illness, and farm management differences were not controlled and might have influenced measured IgG concentrations. Because enrollment was based on owner consent and veterinarian availability rather than consecutive or random sampling, spectrum bias cannot be excluded. The distribution of FTPI severity in this cohort might therefore differ from that of a general screening population, affecting estimates of diagnostic sensitivity and specificity. The wide CIs observed for sensitivity estimates, particularly for complete FTPI, reflect the limited number of positive cases in this study and indicate reduced precision of these estimates. This limitation should be considered when interpreting the reported diagnostic performance. Diagnostic performance was evaluated using matrix-specific datasets with independent observations (1 measurement per foal), which reduces bias related to within-animal correlation; however, the limited number of positive cases within each dataset resulted in wide CIs for some estimates. Based on the exploratory nature of this diagnostic validation study, formal hypothesis-testing power calculations were not performed. However, assuming a sensitivity of 0.90-0.95 at the clinically relevant < 400 mg/dL cutoff, approximately 35-75 FTPI-positive samples would be required to achieve a 95% CI half-width of ±10% to ±5% in a future confirmatory study. In addition, this study was not designed to validate LFIA performance across the full range of IgG concentrations, particularly at high IgG values indicative of adequate passive immunity. This study therefore focused primarily on assay performance at clinically actionable FTPI thresholds, reflecting the value of point-of-care IgG testing for rapid identification of foals at risk of FTPI. These limitations reflect practical and ethical constraints inherent to field-based studies in neonatal foals and should be considered when extrapolating the results to other populations.

In conclusion, the Vcheck foal IgG test offers a practical and rapid option for point-of-care assessment of passive immunity in neonatal foals. Its agreement with RID for FTPI classification and its analytical precision support its use as a screening tool for early identification of foals that could benefit from veterinary intervention.

## Data Availability

The data supporting the findings of this study are available from the corresponding author upon reasonable request.

## Off-label antimicrobial declaration

The authors declare no off-label use of antimicrobials.

## Institutional animal care and use committee or other approval declaration

This study was approved by the Institutional Animal Care and Use Committee (IACUC) of the Korea Racing Authority (approval No.: KRA IACUC-2308). All procedures were performed according to the IACUC guidelines. Owners provided informed consent for the use of all study data.

## Appendix: Detailed materials and methods

This study was designed and reported in accordance with the STARD (Standards for Reporting of Diagnostic Accuracy Studies) guidelines for diagnostic test evaluation (Figure 1). Radial immunodiffusion was used as the reference standard for primary method comparison and agreement analyses, whereas diagnostic performance analyses were conducted separately for each sample matrix (serum, plasma, and whole blood) using independent observations within each dataset. The ELISA was included only as a secondary clinical comparator.

### Animals

Mares had a mean age of 11 years (range 4-19 years). Mares were provided water ad libitum and were fed hay and an individually apportioned commercial concentrate. During the day, mares were turned out to graze with other mares and at night they were housed in individual stalls. Foals had unrestricted access to the dam’s udder and no supplements were provided. As farms did not routinely measure serum immunoglobulin G (IgG) in foals or assess colostrum quality, no interventions such as colostrum supplementation or plasma transfusion were administered before blood collection.

Samples were collected during routine veterinary visits conducted by 2 veterinarians. Each foal’s owner provided consent to participate with the understanding that they would be notified of the test results if complete or partial failure of transfer of passive immunity (FTPI) was identified.

Foals were enrolled on a convenience basis, determined by owner consent and veterinarian availability at a limited number of commercial breeding farms. Consecutive or random enrollment was not feasible due to the unpredictable timing of foaling and the requirement for blood sampling within 24 h of birth in privately owned animals. No selection criteria based on perceived FTPI risk or farm management practices were applied.

A total of 42 foals were enrolled from 18 commercial breeding farms. All foals were Thoroughbreds and were sampled at 1 day of age. The study population comprised 20 colts and 22 fillies. Only foals with no apparent clinical abnormalities at the time of routine veterinary visits were sampled. Confirmation of colostrum ingestion was obtained for all foals based on farm records and owner reports. However, colostrum quality, volume, and exact timing of ingestion were not routinely recorded.

Samples with lateral flow immunoassay (LFIA) results outside the analytical range of the assay (<100 or > 1000 mg/dL) were prospectively excluded from primary method comparison and agreement analyses.

### Samples

This study was planned in advance to evaluate LFIA performance across clinically relevant sample matrices used in point-of-care settings, namely, serum, plasma, and whole blood. Accordingly, all 3 sample types were collected and analyzed. Sample collection priorities were prespecified during study design in the following order: serum, whole blood, and plasma. Serum was prioritized because it is the required matrix for the reference method, radial immunodiffusion (RID). Whole blood was collected as an immediately applicable matrix for point-of-care testing, whereas plasma was included only when sufficient volume could be obtained after anticoagulation and additional centrifugation. Plasma was therefore treated as a secondary sample matrix, reflecting practical constraints of field-based sample processing.

Blood samples were collected within 24 h of birth from the jugular vein into 6 mL BD Vacutainer EDTA tubes and 5 mL BD Vacutainer Serum Separating Tubes II Advance Tube (SST) (Becton, Dickinson and Company, Franklin Lakes, NJ, USA). Samples were transported to the laboratory on ice. Plasma from the BD Vacutainer EDTA tubes was used for the ELISA assay and the remaining plasma was stored at −80°C.

Blood collected in the SST tubes was centrifuged at 4000 *g* for 20 min. Serum was transferred to 5.0 mL Eppendorf tubes and stored −80°C for subsequent IgG analysis. The frozen serum samples were shipped as a single batch on ice to the laboratory for RID (RID test for equine IgG, product number 828411, Triple J Farms, Bellingham, WA, USA) and LFIA testing.

### Laboratory analyses

Immunoglobulin G concentrations were determined using the SNAP-based immunoassay, LFIA, and RID, following the manufact-urer’s instructions. SNAP ELISA testing was performed on the day of blood collection. Serum samples were subsequently frozen and transported to the laboratory, where LFIA and RID testing were performed on the same day within 1 week of sample collection.

#### ELISA

Immunoglobulin G was assessed using the SNAP foal IgG test (IDEXX Laboratories Inc, Westbrook, ME, USA) according to the manufacturer’s instructions. A sample of whole blood was mixed with the sample diluent using a disposable loop. After discarding the first 5-10 drops from the bottle, one drop of the diluted sample was applied to the sample spot, followed by the addition of the conjugate to the device, which was then activated.

After 7 min of color development, the test was interpreted by 2 independent observers using visual comparison of the sample spot with the 2 calibration spots provided by the manufacturer. Results were categorized as < 400, 400-800, or > 800 mg/dL. Both observers evaluated the same test results to minimize interpretive bias and SNAP results were recorded before RID testing.

#### LFIA

The Vcheck foal IgG test (BioNote Inc, Korea) is a fluorescence-based lateral flow immunoassay (LFIA) designed for quantitative measurement of equine IgG in serum, plasma, or whole blood. The assay utilizes specific anti-equine IgG antibodies conjugated to fluorescent labels. Upon application of the diluted sample, the IgG in the specimen forms immunocomplexes that migrate along a nitrocellulose membrane and bind to immobilized antibodies, producing a fluorescent signal proportional to the IgG concentration.

Sample preparation and testing were performed according to the manufacturer’s instructions. A total of 5 μL of serum or plasma (or 10 μL of whole blood) was added to a 10 mL assay diluent vial and mixed thoroughly by inversion. A 100 μL aliquot of the diluted sample was then applied to the sample port of the test cartridge. The device was immediately inserted into the Vcheck V200 Analyzer and the test was initiated. After 5 min, the analyzer provided a quantitative IgG concentration ranging from 100 to 1000 mg/dL. Readings below or above this range were displayed as “<100” or “>1000 mg/dL,” respectively.

The assay was performed at room temperature (15-30°C) and all reagents were equilibrated before use. Results were interpreted using the following thresholds: < 400 mg/dL (complete FTPI), 400-800 mg/dL (partial FTPI), and > 800 mg/dL (adequate passive immunity). These cut-offs were selected to reflect common clinical decision thresholds rather than to define mutually exclusive or hierarchical categories.

#### Radial immunodiffusion assay (RID)

The radial immunodiffusion (RID) assay (Triple J Farms, USA) was used as the reference method for IgG quantification. This method is based on antigen–antibody precipitation in agarose gels embedded with anti-equine IgG antibodies.

Whole blood was collected into plain tubes at field sites and allowed to clot at ambient temperature. Samples were transported upright to the laboratory under cooled conditions (2-8°C) and processed within 2-3 h of arrival. According to the manufacturer’s instructions (Triple J Farms Manual, Product no. 828411), samples were centrifuged at approximately 200 RCF to separate serum from the clot and cellular components and the serum supernatant was collected for RID testing. Radial immunodiffusion results were interpreted by laboratory personnel blinded to the LFIA and ELISA results.

Test procedure: 5 μL of each serum sample was dispensed into individual wells of the RID plate. Reference standards (low, medium, and high IgG concentrations) were included on each plate. Plates were incubated upright at room temperature (20-24°C) for 18-24 h to allow antigen diffusion and precipitation ring formation.

Ring diameters were measured to the nearest 0.1 mm and concentrations were determined from the calibration curve derived from the reference standards. Results were reported in mg/dL. If a sample ring exceeded the highest standard, the sample was diluted and retested.

To assess the reliability of the RID assay, inter-reader reliability, and intra-assay repeatability were evaluated using intraclass correlation coefficients (ICCs). Because RID-derived IgG concentrations represent continuous data, ICC was selected as the appropriate metric of agreement. For inter-reader reliability, RID plates were independently interpreted by 3 readers and IgG concentrations were calculated from measured ring diameters. For intra-assay repeatability, the same samples were measured in triplicate under identical conditions. Agreement was quantified using a 2-way random-effects model with absolute agreement. Intraclass correlation coefficient values were interpreted according to conventional criteria, with values > 0.90 indicating excellent reliability.

### Linearity assessment

To evaluate the linearity of the LFIA, high IgG serum samples (RID > 2000 mg/dL) were selected and serially diluted at 1:2, 1:3, 1:4, 1:6, 1:8, and 1:16. Each dilution was tested in duplicate. The expected IgG values based on dilution ratios were compared with LFIA-measured values. Linear regression analysis was performed and the coefficient of determination (*R*^2^) was calculated to assess the linearity of the LFIA within its analytical range (100-1000 mg/dL).

### Precision analysis

Precision was evaluated in terms of intra-assay and inter-assay variability. Four serum samples were prespecified to target IgG concentrations approximately evenly distributed across the analytical range of the LFIA (approximately 200, 400, 800, and 1000 mg/dL). Based on available sample material, the closest achievable IgG concentrations were 226, 432, 775, and 1005 mg/dL. Intra-assay precision was assessed using 3 replicate measurements within a single analytical run on each of 5 nonconsecutive days and inter-assay precision was calculated using daily mean values across the 5 days. The coefficient of variation (CV%) was calculated for each concentration level.

### Accuracy analysis

Analytical accuracy of the LFIA was evaluated by comparing IgG concentrations measured by LFIA with RID results as the reference standard. To avoid nonindependence of observations arising from repeated measurements within the same foal, all primary accuracy and agreement analyses were restricted to serum samples.

Diagnostic performance analyses (sensitivity and specificity) were conducted separately for each sample matrix (serum, plasma, and whole blood), using only independent observations within each dataset. Each matrix-specific dataset included one measurement per foal and was analyzed independently. No pooling across sample types was performed, and diagnostic performance estimates were therefore derived at the foal level within each matrix-specific dataset.

Pearson correlation analysis and linear regression were used to assess the association between methods and to derive the coefficient of determination (*R*^2^) and regression equation. Agreement between LFIA and RID measurements was further evaluated using Bland–Altman analysis.

A regression of the differences (LFIA − RID) on the measurement mean was performed to formally assess proportional bias. A non-zero slope would indicate concentration-dependent bias. Given evidence of heteroscedasticity, agreement was additionally evaluated using a log-transformed (ratio-based) Bland–Altman analysis.

### Categorical agreement analysis

Categorical agreement was assessed using Cohen’s κ statistic. Ninety-five percent CIs for the unweighted κ were calculated using the asymptotic standard error based on observed and expected agreement proportions. Because IgG concentration categories are ordinal, linear, and quadratic weighted κ statistics were also calculated.

### Sensitivity and specificity

To evaluate diagnostic performance at clinically relevant decision points, sensitivity and specificity of the LFIA were calculated using RID as the reference standard at 2 prespecified IgG thresholds. These thresholds were < 400 mg/dL, corresponding to complete failure of transfer of passive immunity (FTPI) and ≤ 800 mg/dL, corresponding to partial or complete FTPI. Samples were dichotomized for analysis based on RID into: (1) complete FTPI (<400 mg/dL) and (2) partial or complete FTPI (≤800 mg/dL).

Analyses were performed separately for each sample matrix (serum, plasma, and whole blood), with each dataset comprising independent observations (1 measurement per foal). No pooling of measurements across matrices was performed. Ninety-five percent CIs for sensitivity and specificity were estimated using the Wilson method.

### Receiver operating characteristic (ROC) analysis

Receiver operating characteristic curve analysis was conducted to evaluate the diagnostic accuracy of the LFIA in identifying complete FTPI (<400 mg/dL) and partial or complete FTPI (≤800 mg/dL). The area under the ROC curves was calculated for the LFIA at the < 400 and ≤ 800 mg/dL cutoffs. Ninety-five percent CIs for ROC AUCs were estimated using the DeLong method.

### Statistical analysis

Statistical analyses were performed using open source Python (version 3.7.12), with statistical significance set at *P* < .05. Results were presented as mean ± SD and descriptive statistics were used to summarize the data. Because this study was designed as an exploratory validation study, no formal prospective power calculation was performed. Sample enrollment was further constrained by the requirement for owner consent to collect blood samples from foals within 24 h of birth. All statistical analyses were performed by the academic authors, who had full access to all study data and were responsible for the integrity of the data and accuracy of the analysis.

Normality of continuous variables was assessed before analysis using the Shapiro–Wilk test and visual inspection of histograms and Q–Q plots. Radial immunodiffusion IgG concentrations showed a borderline deviation from normality, whereas LFIA measurements did not significantly deviate from normality. Normality of the method differences (LFIA − RID) and regression residuals was evaluated to confirm assumptions underlying Bland–Altman and regression analyses and both were normally distributed.

Given the borderline deviation from normality observed for RID IgG concentrations, agreement was additionally evaluated using a log-transformed (ratio-based) Bland–Altman approach to appropriately account for nonconstant variability across the measurement range.

## Abbreviations

AUC: area under the curve
FTPI: failure of transfer of passive immunity
ICC: intraclass correlation coefficient
LFIA: lateral flow immunoassay
RID: radial immunodiffusion
ROC: receiver operating characteristic
TIA: turbidimetric immunoassay
